# Comparative genomics of Ascetosporea gives new insight into the evolutionary basis for animal parasitism in Rhizaria

**DOI:** 10.1186/s12915-024-01898-x

**Published:** 2024-05-03

**Authors:** Markus Hiltunen Thorén, Ioana Onuț-Brännström, Anders Alfjorden, Hana Pecková, Fiona Swords, Chantelle Hooper, Astrid S. Holzer, David Bass, Fabien Burki

**Affiliations:** 1 Department of Organismal Biology, Uppsala University, Norbyv. 18D, Uppsala, SE-752 36 Sweden; 2 Present Address: Department of Ecology, Environment and Plant Sciences, Stockholm University, Svante Arrhenius V. 20 A, Stockholm, SE-114 18 Sweden; 3 Present Address: The Royal Swedish Academy of Sciences, Stockholm, SE-114 18 Sweden; 4 Present Address: Department of Ecology and Genetics, Uppsala University, Norbyv. 18D, Uppsala, SE-752 36 Sweden; 5 Present Address: Natural History Museum, Oslo University, Oslo, 0562 Norway; 6 Institute of Parasitology, Biology Centre of the Czech Academy of Sciences, Branišovská 31, České Budějovice, 370 05 Czech Republic; 7 Marine Institute, Rinville, Oranmore, H91R673 Ireland; 8 Centre for Environment, Fisheries and Aquaculture Science (Cefas), Weymouth Laboratory, Weymouth, Dorset, DT4 8UB UK; 9 Sustainable Aquaculture Futures, Biosciences, University of Exeter, Stocker Road, Exeter, EX4 4QD UK; 10 Division of Fish Health, University of Veterinary Medicine, Veterinärplatz 1, Vienna, 1210 Austria; 11 Natural History Museum (NHM), Science, London, SW7 5BD UK; 12 Science for Life Laboratory, Uppsala University, Uppsala, Sweden

**Keywords:** Genome reduction, Reductive evolution, Evolutionary transition, Phylogeny, Protozoa, Intracellular parasite, *Bonamia*, *Marteilia*, *Paramarteilia*, *Mikrocytos*, *Paramikrocytos*

## Abstract

**Background:**

Ascetosporea (Endomyxa, Rhizaria) is a group of unicellular parasites infecting aquatic invertebrates. They are increasingly being recognized as widespread and important in marine environments, causing large annual losses in invertebrate aquaculture. Despite their importance, little molecular data of Ascetosporea exist, with only two genome assemblies published to date. Accordingly, the evolutionary origin of these parasites is unclear, including their phylogenetic position and the genomic adaptations that accompanied the transition from a free-living lifestyle to parasitism. Here, we sequenced and assembled three new ascetosporean genomes, as well as the genome of a closely related amphizoic species, to investigate the phylogeny, origin, and genomic adaptations to parasitism in Ascetosporea.

**Results:**

Using a phylogenomic approach, we confirm the monophyly of Ascetosporea and show that Paramyxida group with Mikrocytida, with Haplosporida being sister to both groups. We report that the genomes of these parasites are relatively small (12–36 Mb) and gene-sparse (~ 2300–5200 genes), while containing surprisingly high amounts of non-coding sequence (~ 70–90% of the genomes). Performing gene-tree aware ancestral reconstruction of gene families, we demonstrate extensive gene losses at the origin of parasitism in Ascetosporea, primarily of metabolic functions, and little gene gain except on terminal branches. Finally, we highlight some functional gene classes that have undergone expansions during evolution of the group.

**Conclusions:**

We present important new genomic information from a lineage of enigmatic but important parasites of invertebrates and illuminate some of the genomic innovations accompanying the evolutionary transition to parasitism in this lineage. Our results and data provide a genetic basis for the development of control measures against these parasites.

**Supplementary Information:**

The online version contains supplementary material available at 10.1186/s12915-024-01898-x.

## Background

Parasites have played critical roles in the evolutionary history of virtually all forms of cellular life, and parasitism has arisen countless times during evolution [1]. The evolutionary transition from a free-living lifestyle to parasitism presents a multitude of challenges, such as developing effective mechanisms for infection and to evade host defense. Other selective pressures acting on the parasite may be relieved, e.g., of conservation of some metabolic pathways once resources can instead be scavenged from the host [2]. Especially in obligate, intracellular parasites, the resulting phenotypic effects may be profound, taken to the extreme in lineages such as Microsporidia, where considerable reductions in cellular complexity, physiology, and metabolic potential have evolved [3, 4]. Consequently, the genomes of intracellular parasites tend to be reduced, with extensive gene loss and reduction in number and length of introns and intergenic spaces, along with reduced mitochondrion-related organelles [5, 6]. While genome reduction may be the most prevalent evolutionary trend for intracellular parasites, most groups also showcase genomic innovation to adapt to host environment [7–9]. Furthermore, parasites often exhibit elevated rates of speciation and molecular evolution, which may be due to a smaller effective population size caused by sequential bottlenecks during transmission, a higher mutation rate to be competitive in the evolutionary arms-race with the host, fewer outcrossing opportunities, or a combination of these factors [1, 10, 11].

Ascetosporea is a class of eukaryotic minute intracellular parasites that belong to Endomyxa in the large and diverse supergroup Rhizaria. These single-cell parasites predominantly infect invertebrates, such as arthropods and bivalves, in marine and freshwater environments [12–14]. They include relatively well-known parasites such as *Mikrocytos mackini*, the causative agent of Denman Island disease in Pacific oysters, *Paramikrocytos canceri*, infecting the edible crab *Cancer pagurus*, and *Marteilia refringens* and *Bonamia ostreae*, both infecting European flat oysters that have been attributed to large population declines in the 1970–1980s [15, 16]. However, environmental surveys and targeted molecular probing have revealed a much higher diversity of Ascetosporea than previously suspected, infecting a wide range of invertebrate hosts [13, 17–22]. Five orders are currently recognized in Ascetosporea: the long-time members Haplosporida and Paramyxida, morphologically united by their intricate spore ornamentation [12], and Claustrosporida, Paradinida, and Mikrocytida added more recently [18, 23, 24]. However, the phylogenetic relationships among these groups, and the position of Ascetosporea within Rhizaria, remain to be established [12, 18, 25].

Despite being of high ecological and socio-economic importance, limited genomic information exists on Ascetosporea. This can be primarily explained by difficulties in sample acquisition, due to the small sizes and intracellular nature of these parasites, and the lack of cultures [12]. Recently, the genomes and transcriptomes of the mikrocytids *M. mackini* and *Paramikrocytos canceri* have been analyzed [26–28]. These studies revealed that they have followed a similar evolutionary path as in other unrelated intracellular parasites such as Microsporidia, notably by evolving a highly reduced mitochondrion and low functional coding capacity in general, while having an extremely high rate of molecular evolution [26, 27]. However, the generality of these findings for other ascetosporean groups remains to be investigated, especially because genome data is lacking for other members of the class and no close free-living outgroup has been identified and sequenced.

Here we report the first genome assemblies for three ascetosporeans belonging to two orders: *B. ostreae* (Haplosporida), *Marteilia pararefringens*, and *Paramarteilia canceri* (Paramyxida), in addition to that of the undescribed Cercozoa sp. strain M6MM (NCBI: txid1215613; hereafter referred to as M6MM). M6MM is an amphizoic amoeba that we hypothesized to be a close relative to Ascetosporea based on sequence similarity of the 18S ribosomal RNA gene (NCBI GenBank: JQ271793.1). We supplemented these new data with the genomes of *M. mackini* [28], and *Paramikrocytos canceri* (European Nucleotide Archive: ERZ16272710) [27]. Using this dataset, we carried out a comprehensive phylogenomic analysis of Rhizaria including Ascetosporea and show that this group of parasites is sister to Retaria and Apofilosa (*Gromia, Filoreta*), with elevated evolutionary rates especially in the Paramyxida-Mikrocytida clade. Furthermore, our analyses placed M6MM in Apofilosa as sister taxon to *Gromia* and *Filoreta*. We show that Ascetosporean genomes have undergone functional reduction, primarily in metabolic pathways, and expansions in gene families encoding proteases and transporters. Together, our results present important new genomic information from a lineage of enigmatic parasites, shedding new light on the free-living to parasite evolutionary transition in Rhizaria.

## Results and discussion

### Assembling and decontaminating ascetosporean genomes

Ascetosporean parasites infect a range of invertebrate hosts, including oysters, mussels, and crustaceans [13, 15, 16, 29]. Spending most or all of their known life cycles as intracellular parasites, it is challenging to acquire molecular data from ascetosporean species [12]. To overcome issues with obtaining pure isolates of parasite cells for genome sequencing, we bioinformatically retrieved data from the targeted organisms using metagenomes obtained from host infected tissues as previously described [27]. Briefly, this method relies on sequencing both infected and parasite-free host tissue in different libraries, in order to apply in silico decontamination to remove host sequences that have high similarity to the parasite-free metagenome. Contigs were identified based on differential GC%, depth of read coverage, and/or similarity to known sequences in databases, including the newly produced parasite-free host tissues. To include all currently available ascetosporean genomes in our analyses, the genomes of *Paramikrocytos canceri* [27] and *M. mackini* [28] (re-assembled and annotated using the same methodology as for the new genomes) were added. After several consecutive rounds of contamination screening, filtering of reads, and re-assembly, final genome assemblies of the target ascetosporean parasites were produced. As in any metagenomic analysis, this method is subject to a trade-off between minimizing false positive gene calls (i.e., from contaminants) and false negatives (i.e., filtering out genes from the target organisms). As a general rule, we opted in favor of stringency during cleaning, which resulted in low detectable contamination (Additional file 1: Fig. S1) but at a potential cost of also removing genuine genes for the target organisms. After submission of annotated genomes to the NCBI genome database, 0.6%, 0.1%, 0.04%, and 4.7% of genes in M6MM, *M. pararefringens*, *Paramarteilia canceri*, and* B. ostreae*, respectively, were identified as putative remaining contaminants by the NCBI Foreign Contamination Screen [30] (see “Methods” for details).

To assess assembly quality, we used comparisons of k-mer frequencies between raw reads and assemblies, which revealed that almost all information from the raw reads was captured in the assemblies (Additional file 2: Fig. S2). This analysis also suggested that most genomes were haploid, showing a single peak of unique k-mer numbers, except for *Paramarteilia canceri* and *Paramikrocytos canceri*, where two peaks were found, indicating diploidy (Additional file 2: Fig. S2) [27]*.* Completeness assessment by BUSCO [31] revealed relatively low scores in our genome assemblies, ranging from 33.4 to 59.1% complete and fragmented BUSCOs (Additional file 3: Fig. S3). Low BUSCO scores are expected for parasites such as those studied here, which have a reduced gene content, few close relatives in sequence databases, and are fast-evolving, complicating orthology assessment [28, 32]. It is also likely that some genes are missing in our assemblies due to low coverage of parasite data, for example for *B. ostreae* (Additional file 2: Fig S2). Conversely, BUSCO score can be inflated if assessed before thorough cleaning; e.g., the high BUSCO completeness recently reported for the *B. ostreae* transcriptome [33] may be driven partly by undetected contaminating sequences (Additional file 4: Fig. S4). As an alternative to BUSCO, we also compared the presence of a set of conserved housekeeping genes (126 genes) previously used in phylogenomic studies of Ascetosporea [26, 27]. We found homologs of the majority of genes in this set in our gene annotations (Table 1).

**Table 1:** Genome assembly and annotation statistics

	M6MM	*Bonamia ostreae*	*Paramikrocytos canceri*	*Mikrocytos mackini*	*Marteilia pararefringens*	*Paramarteilia canceri*
Assembly size (Mb)	23.67	12.24	12.63	15.44	22.37	36.83
# Scaffolds	2244	9705	3113	5841	3572	14,859
Assembly N50 (bp)	23,794	1417	6806	5394	12,642	3776
G + C content (%)	52.8	33.3	30.1	32.7	30.7	23.9
Coding content (%)	56.3	32.1	16.6	28.7	22.1	8.1
Repeat content (%)	9.82	4.33	18.45	37.09	24.49	22.3
Gene models	9694	5253	2340	5260	5208	4941
Mean CDS length (bp)	1182	658	912	799	874	611
Median intergenic distance (bp)	176.5	218	1615	241	1050	500
Mean introns per mRNA	10	2	0	0	2	1
Presence of genes from [26] (%)	95.2	65.9	89.7	98.4	73.8	65.1

We opted here to re-assemble the genome of *M. mackini* following the same pipeline as for the other genomes, which resulted in a much smaller assembly size compared to the available assembly (15.4 versus 49.7 Mb) [28]. This large size difference is likely caused by differences in how repetitive genomic regions were handled during assembly, and mikrocytid genomes are known to carry a high proportion of transposable elements [27, 28], complicating genome assembly and annotation. The actual genome size of *M. mackini* is difficult to estimate without further data, such as long-read sequencing or flow cytometry, and likely resides somewhere in between these two estimates. To further evaluate the genome size difference, we compared our gene annotations to the available data for *M. mackini* but also the other mikrocytid *Paramikrocytos canceri* [26–28]. In both cases, our new assemblies contained fewer gene models (5260 vs 14,372 in *M. mackini*; 2340 vs 8201 in *Paramikrocytos canceri*). A potential explanation for these differences is our more stringent filtering of gene models residing in repetitive regions or without having the support of mapped transcriptomic reads. To assess this possibility, we mapped the protein sequences from the previously available annotations to the newly generated genome assemblies and applied the same filters as during our new annotations. After this, 198 and 767 gene models remained in *M. mackini* and *Paramikrocytos canceri*, respectively. To summarize, despite the inherent difficulties in obtaining high-quality molecular data for uncultured intracellular parasites such as Ascetosporea, the method we used for genome assembly and decontamination resulted in low contamination levels with relatively high completeness (Table 1).

### Few genes and abundant non-coding sequences in ascetosporean genomes

The decontaminated genome assemblies varied in size between 12 and 36 Mb (Table 1). Genomic GC content was found to be lower in all parasites compared to the amphizoic amoeba M6MM (24–33% versus 53%, respectively; Table 1). All genome assemblies were subjected to structural and functional gene annotation, revealing that the parasites overall carried fewer predicted genes (between 2340 and 5253) compared to M6MM (9694 predicted genes; Table 1). Canonical spliceosomal introns were found in all genomes except in *M. mackini* and *Paramikrocytos canceri*, where only few and very short introns were predicted, confirming previous observations in these species [28]. The genomes of all parasites carried a high amount of non-coding sequence (72–92%). Intergenic distances were especially long in *Paramikrocytos canceri*, with a median of 1615 bp (Table 1). Non-coding sequences in all studied organisms were partly occupied by genomic repeats (~ 4–37%). The function of the remaining non-coding and non-repetitive sequence (36–70% in the parasites), if any, is unclear, but it is possible that remnants of highly diverged pseudogenes and/or transposons reside in these regions. The amount of non-coding sequence stands out against results from Microsporidia, where redundant sequences tend to be lost, resulting in much more compact genomes in addition to reduction [28, 34].

Following annotation of gene functions, 75.7% of the predicted mRNAs in M6MM were annotated, whereas in the ascetosporean parasites between 58.6 and 70.0% of mRNAs received annotation. Functional annotation also revealed that *M. mackini* carried a group of genes (346 genes) with putative *Mutator-*like (MULE) transposase domains [35]. Reasoning that these genes are likely encoded by transposable elements that escaped repeat annotation, we excluded them from downstream analyses.

The mikrocytids *Paramikrocytos canceri* and *M. mackini* are known for their reduction in cellular complexity, which includes loss of most spliceosomal introns, reduction of mitochondria, loss of the mitochondrial genome, and loss of many metabolic functions [26–28]. Our newly sequenced ascetosporean genomes did not display the same level of extreme reduction as the mikrocytids. Both paramyxids (*M. pararefringens* and *Paramarteilia canceri*) and the haplosporidian *B. ostreae* carry canonical introns. In *B. ostreae*, a putative partial mitochondrial genome was recovered in a single scaffold (“scaffold_2”; ~ 21 kb), encoding ribosomal RNA genes, transfer RNAs, and subunits of ATPase, cytochrome *c* oxidase, and cytochrome b (Additional file 5: Datafile S1). In the paramyxids, however, no clear mitogenomes could be recovered. The mitogenomes in many protists, including rhizarians, are known to be replicated by the nuclear-encoded plant and protist organellar DNA polymerase (POP) [36]. We were able to identify putative homologs of this gene in *B. ostreae* and M6MM (e-values < 1e − 29), but not in the other ascetosporeans. While cytological results suggest that paramyxids have retained mitochondria, several authors have noted that they are few and appear degenerate, e.g., in the complexity of cristae [37, 38]. The lack of mitogenomes in our paramyxid assemblies could thus be explained by a low number of mitochondria in the isolated cells, a reduced or lost mitogenome, or a combination of both factors. The apparent lack of the POP type polymerase supports the lack of mitogenomes but it is also possible that this gene is too divergent to allow homology detection.

### Ascetosporea is monophyletic and sister to Apofilosa and Retaria

**Fig. 1:**
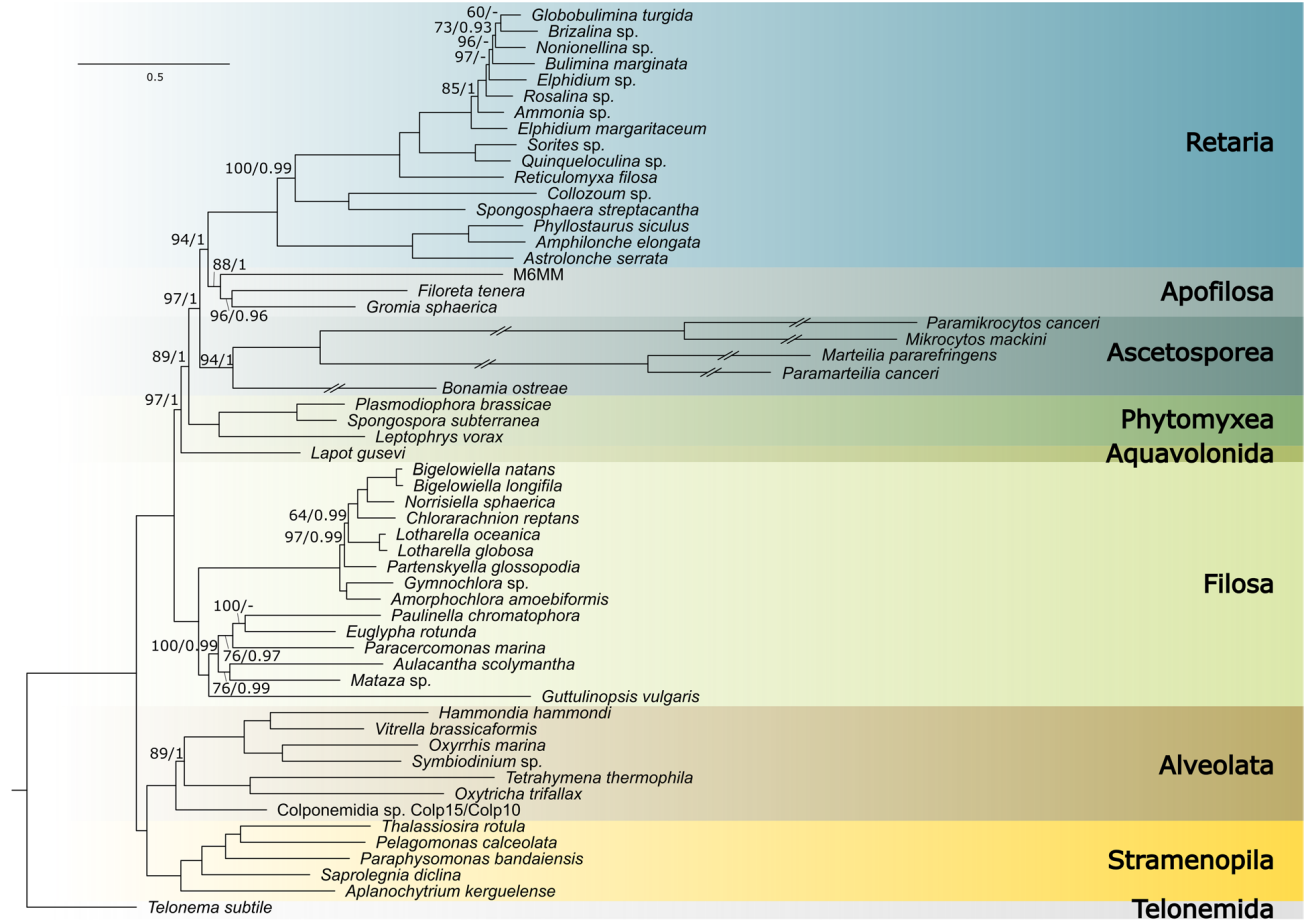
Phylogeny of Rhizaria. The tree was built using maximum likelihood from a concatenated supermatrix of 225 single-copy orthologs with the LG + C60 + G + F model in IQ-TREE2. Branch values depict from left to right ultra-fast bootstrap support and posterior probability scores from the consensus of three converged chains of CAT + GTR + GAMMA in PhyloBayes. Branches without values are fully supported by both analyses. The PhyloBayes analysis was performed on a subsampled matrix where the 38% slowest-evolving sites were retained. Branch lengths scaled to substitutions per site, as per the scale bar at the bottom. Within Ascetosporea, branches were collapsed to half their length. For details about the source data of included taxa, see Additional file 6: Dataset S2

Ascetosporea consists of five orders (Mikrocytida, Haplosporida, Paramyxida, Paradinida, Claustrosporida), but the relationships between them, as well as their placement in a broader phylogenetic context, remain unclear [12, 39]. Here, we used the first genome data for Paramyxida and Haplosporida, together with available data from Mikrocytida, to provide an insight into the evolutionary history of these three important Ascetosporean orders. Importantly, our dataset also includes genome data for M6MM, a lineage possibly related to Ascetosporea based on an initial 18S rRNA tree (not shown). Using rigorous phylogenetic analyses, we compiled a set of 225 orthologous single-copy genes based on previous work [40, 41]. Our taxon-sampling contained a total of 56 taxa, including M6MM, the five ascetosporean species, other rhizarians with publicly available genomes or transcriptomes, and outgroups consisting of *Telonema subtile* and selected alveolates and stramenopiles (Additional file 6: Datafile S2). All included genes were thoroughly investigated for paralogy and contamination by manual inspection of alignments and single-gene trees with representatives from all known major groups of eukaryotes and some bacteria, prior to concatenating gene alignments. The resulting supermatrix consisted of 67,785 amino acid sites (42% overall missing data). This supermatrix was subjected to Maximum Likelihood (ML) phylogenetic reconstruction by IQ-TREE2 [42] with LG + C60 + G + F as the substitution model, selected by ModelFinder [43]. This analysis recovered Ascetosporea as a monophyletic group (UFboot = 94%, PP = 1), in a sister position to an assemblage composed of Apofilosa (containing the filose amoebae *Gromia*, *Filoreta*, and M6MM) and Retaria (containing Foraminifera, and the radiolarian Acantharea and Polycystinea) (Fig. 1). Within Ascetosporea, Paramyxida and Mikrocytida branched together (UFboot = 100%, PP = 1), while Haplosporida was sister to this group. This topology is consistent with previous analyses of internal relationships within Ascetosporea based on fewer genes [17, 18, 26, 39].

As is apparent in Fig. 1 and known from previous work, Ascetosporea, especially Mikrocytida and Paramyxida, represent extremely fast-evolving eukaryotic taxa [18, 26, 27]. Long branches can be problematic in phylogenetic inferences, so to try to mitigate some of the potential issues caused by, e.g., differential evolutionary rates among sites and lineages, we performed incremental removal of the fastest evolving sites (nine steps of 7000 sites), followed by ML tree reconstruction at each step. The topology was consistent throughout all steps, with gradually increasing ultrafast bootstrap support values at branches of interest, until the dataset became very small (Additional file 7: Fig. S5A). After 42,000 sites were removed (38% of sites left), support was maximal at branches among Ascetosporea, Apofilosa, and Retaria.

We selected this reduced submatrix (42,000 sites removed) for Bayesian phylogenetic inference, using the site heterogeneous CAT + GTR + GAMMA model as implemented in PhyloBayes [44]. We ran three independent chains for > 7000 generations, which reached convergence after a burn-in period of 700 generations (maxdiff < 0.1). The consensus tree of the three chains is shown in Additional file 7: Fig. S5B, which is in agreement with the ML tree (Fig. 1). As another measure of control against long-branch attraction artifacts, we removed the long-branch taxa of Paramyxida and Mikrocytida before running an additional ML analysis of the full supermatrix, which resulted in a similar tree but with lower support values, presumably due to the less complete sequence of *B. ostreae* (Additional file 8: Fig. S6). Finally, we computed a multi-species coalescent tree, using each of the 225 gene alignments as input for IQ-TREE to infer gene trees, and ASTRAL-III to calculate the species tree (Additional file 9: Fig. S7). Overall, these analyses were consistent with the ML tree based on the full dataset (Fig. 1). The order of Apofilosa and Ascetosporea is swapped in the ASTRAL analysis, but support around these branches is low.

The deeper branches in Rhizaria have proven difficult to resolve, with some studies suggesting a grouping of Endomyxa (including Ascetosporea, Apofilosa, and Phytomyxea) with Retaria [25, 45–48], while others favor a monophyletic Cercozoa, including Endomyxa and Filosa, to the exclusion of Retaria [49]. In our analyses, Endomyxa was paraphyletic due to the placement of Retaria close to Ascetosporea, but the support values were not conclusive. Thus, whether Endomyxa represents a real assemblage or not requires further testing, especially as more taxa become available in future studies. More generally, two main parasitic groups are known in Rhizaria: Ascetosporea, which includes only parasites of animals, and Phytomyxea, which contains parasites of plants and algae. Previous work has suggested independent origins of parasitism in these two groups [25], and while our tree differs slightly in topology, it also supports two transitions since each parasitic group is more closely related to free-living lineages.

### Extensive gene loss during ascetosporean evolution

**Fig. 2:**
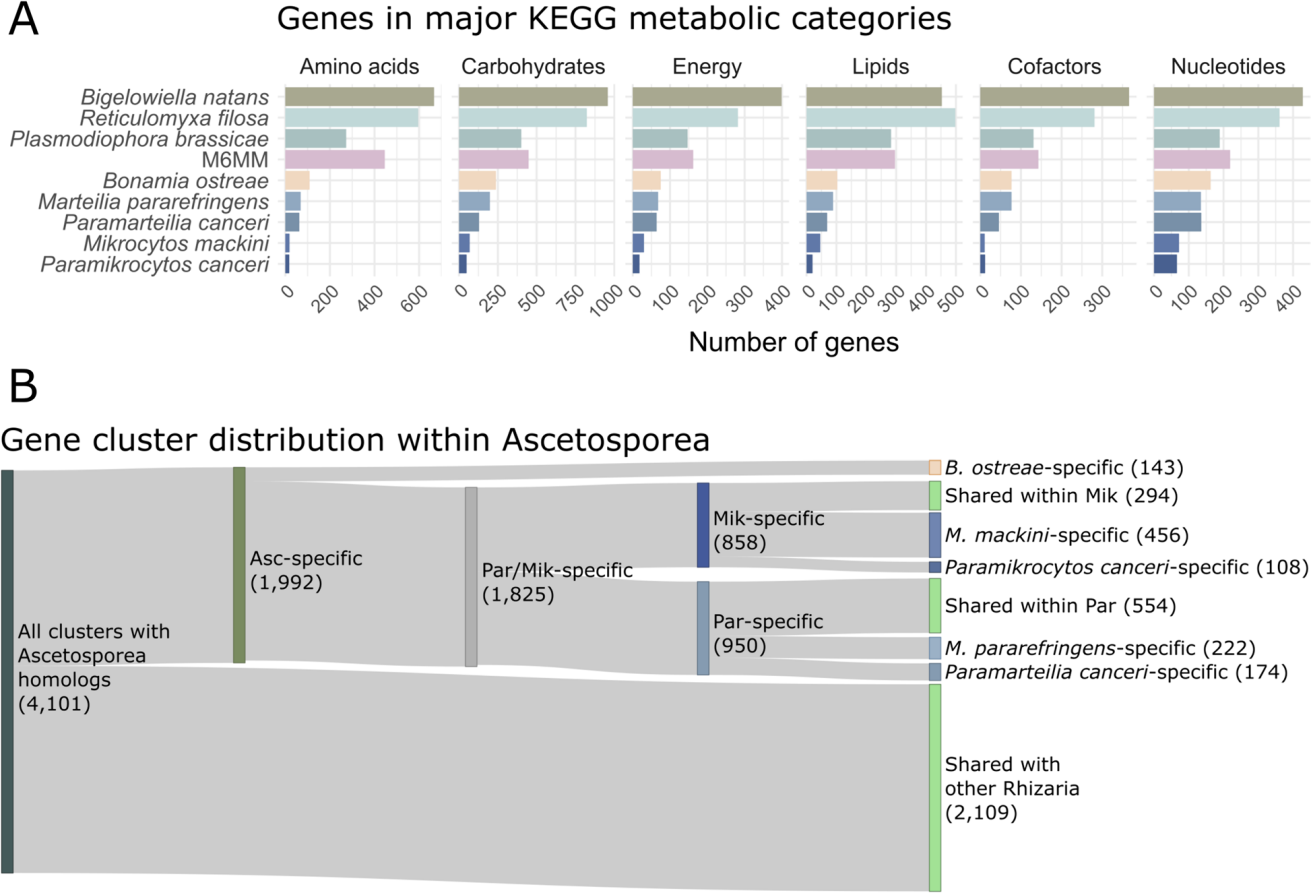
Metabolic genes and gene clusters in Ascetosporea. **A** Number of genes in major categories of metabolic pathways as classified in comparison to the Kyoto Encyclopedia of Genes and Genomes (KEGG). **B** Shared and specific gene clusters. “Specific” refers to clusters that are not found outside that group. Numbers in parentheses refer to the number of clusters in that category. Clusters shared between non-sisters not shown. Asc: Ascetosporea; Par: Paramyxida; Mik: Mikrocytida

We investigated the metabolic repertoire of Ascetosporea (and M6MM) by mapping predicted proteins from each species in our study, along with those of the other Rhizaria with full genome data: *Reticulomyxa filosa* (Retaria), *Plasmodiophora brassicae* (Phytomyxea), and *Bigelowiella natans* (Filosa) [50–52], to major groups of metabolic pathways of the Kyoto Encyclopedia of Genes and Genomes (KEGG). This analysis revealed that the ascetosporean genomes were highly reduced in all metabolic functions, even when compared to the plant parasite *P. brassicae*, while M6MM retained more genes in all functional categories (Fig. 2A). Gene clustering analysis by OrthoFinder [53] of all 103,635 proteins from the nine genomes resulted in 69,750 genes (67%) being classified into 12,313 orthogroups (i.e., gene clusters, or putative families). The remaining 33,885 genes (32.7%) were classified as singletons (i.e., no homolog was predicted in any genome). The number of gene clusters specific to Ascetosporea (i.e., without homologs in *R. filosa*, *P. brassicae*, *B. natans*, or M6MM) was 1992 (16% of all gene clusters), out of which 889 (45% of Ascetosporea-specific clusters) were shared between at least two species (Fig. 2B).

**Fig. 3:**
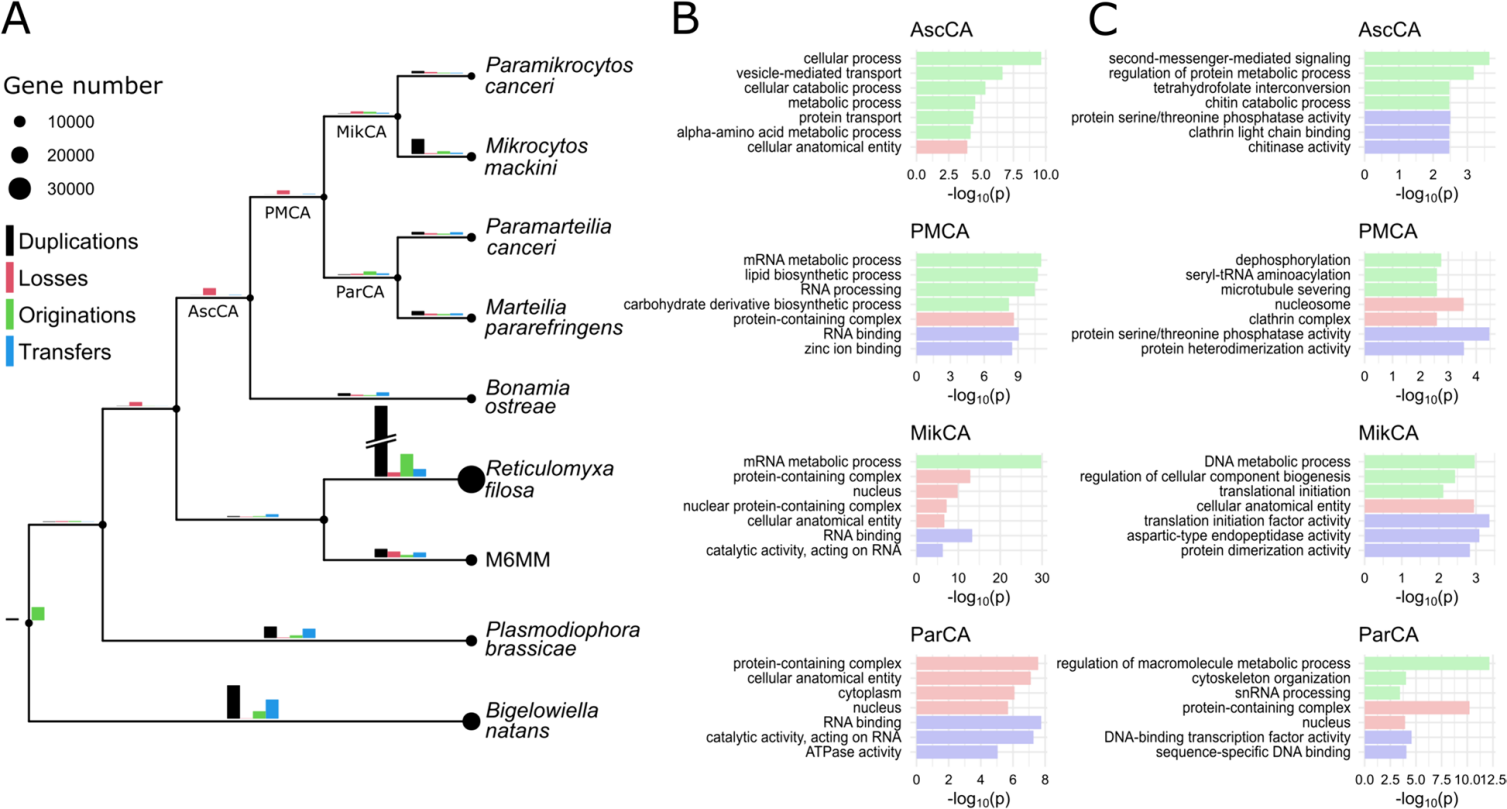
Gene family evolution in Ascetosporea. **A** Gene-tree aware ancestral reconstruction of gene families. Bubble sizes at branches correspond to predicted proteome sizes, and gene evolution events are plotted as bars, where the height of the bar reflects the number of such events (see main text for explanation of the events). The height of the bars in the legend corresponds to 5000 events. The bar for duplications on the branch to *Reticulomyxa filosa* has for plotting purposes been halved in height. **B**, **C** Gene Ontology (GO) enrichment analysis of orthogroups (OGs; i.e., gene clusters) that experienced losses (**B**) and gains (**C**; duplications and originations analyzed together) at ancestral branches within the Ascetosporea sub-tree. For plotting, GO terms have been clustered based on semantic similarity, and one representative term per cluster is shown. Only the seven GO terms with lowest *p*-values are shown, and bars are colored based on top-level GO category (green: Biological Process; red: Cellular Component; blue: Molecular Function). AscCA: Ascetosporea Common Ancestor; PMCA: Paramyxida-Mikrocytida Common Ancestor; MikCA: Mikrocytida Common Ancestor; ParCA: Paramyxida Common Ancestor

To model gene family evolution in Ascetosporea, Amalgamated Likelihood Estimation (ALE) [54] was used based on the gene clusters predicted by OrthoFinder. The events predicted by ALE include gene losses, duplications, originations (i.e., de novo genes or transfers from outside the species tree), and transfers within the species tree. This analysis revealed considerable gene losses at the origin of Ascetosporea and continuing after the split of Haplosporidia, indicating that the transition to parasitism in this group was accompanied by extensive genome reduction (Fig. 3A). We next performed Gene Ontology (GO) Enrichment analysis to investigate if any functional classes of genes were over-represented in reduced gene clusters, i.e., lost more often than expected by chance. At the last common ancestor of Ascetosporea (AscCA), as well as on most internal branches within the group, we found that genes linked to metabolism were over-represented in gene losses (Fig. 3B; Additional file 10: Dataset S3). At AscCA, losses in gene clusters related to protein transport, cilium assembly, and other cellular components were also found. The loss of genes involved in cilium structure and function is interesting as it may have implications for the ability to form flagellated cells, e.g., gametes, during the life cycle. To assess whether Ascetosporea can form flagella, we looked further into the GO term “cilium” (GO:0005929) in our dataset and found that all species have fewer cilium-related genes than any lineage in the outgroups (Additional file 11: Dataset S4). The mikrocytids stood out by having as few as ~ 10 such genes, indicating that they may have lost the ability to form flagella. At the branch representing the common ancestor of Paramyxida and Mikrocytida (PMCA), gene clusters with losses were enriched for RNA splicing, suggesting that reduction of the spliceosome was initiated early during parasitic diversification, despite introns being retained in paramyxids. We also identified some lineage-specific losses. For example, in the common ancestor of mikrocytids (MikCA), reduced gene clusters were found to be involved in heme biosynthesis, mRNA splicing, or encoding mitochondrial membrane proteins. Loss of components in the splicing machinery was expected from previous results [28], while the loss of mitochondrial membrane proteins and heme biosynthesis pathway (initiated in canonical mitochondria [55]) concurs with previous findings showing that mikrocytids possess mitosomes (extremely reduced mitochondria) [26, 27]. Furthermore, the lack of the heme biosynthesis pathway, which is essential in oxygen transport, storage, and metabolism [56], suggests that mikrocytids have evolved the ability to scavenge heme from their hosts, similarly as, e.g., the trypanosomatid *Leishmania* [57]. In the paramyxid common ancestor (ParCA), losses of genes involved in stress response, signal transduction, and organellar components were identified. At the terminal branches of the Ascetosporea clade, functional classes of lost genes largely reflected those at internal branches; though notably, DNA repair genes were over-represented in gene losses in *B. ostreae*, *M. mackini*, and *Paramikrocytos canceri* (Additional file 10: Dataset S3). This finding may indicate a potential link between reduction of the DNA repair machinery and high evolutionary rates in Ascetosporea, reflecting what has been seen in, e.g., Microsporidia and some yeasts [58, 59], although a confounding factor might be difficulty in orthology assessment of highly diverged organisms [60].

In summary, it appears that the transition to parasitism caused a redundancy of many gene families, primarily metabolism-related, leading to their reduction during evolution of Ascetosporea. This result conforms well with what is known about evolution of other parasites [7, 61–63].

### Gene innovations during parasite evolution

While the transition to parasitism in Ascetosporea was primarily characterized by gene loss, some gene clusters increased in size, and these clusters may house functions that were crucial to the evolution of parasitism. Very little gene gain was inferred at the branches AscCA and PMCA, with only few expanding clusters enriched for GO terms such as signaling and phosphatase activity (Fig. 3C). Most gene gains were inferred in more recent ascetosporean evolution, i.e., on terminal branches. In the mikrocytids and their common ancestor (MikCA), gene gains were significantly enriched for, e.g., aspartic-type endopeptidase activity (Fig. 3C). Peptidases are known to play important roles during infection and host evasion for many parasites, thus acting as virulence factors [64]. In apicomplexans, aspartic proteases are implicated during host cell invasion and during degradation of hemoglobin [65, 66]*.* The finding that gene clusters encoding aspartic proteases have expanded during mikrocytid evolution suggests an important function in these parasites as well, possibly during host infection. Another group of expanded clusters in the mikrocytids encoded ATP-binding cassette (ABC) transporters (Fig. 3C). Transmembrane transporters are essential for parasites since they enable nutrient transfer from the host. The *M. mackini* genome was recently reported to contain a variety of transporter proteins [28], and our results indicate that acquisition of such genes started before diversification of Mikrocytida. Also in other parasites, e.g., trypanosomatids, ABC-type transporters are known to have expanded [7].

During paramyxid evolution (ParCA, and *M. refringens* and *Paramarteilia canceri* terminal branches), enriched GO terms in expanded gene clusters included lipid transport (Fig. 3C). Paramyxids are known for their peculiar mitotic process, where newly divided cells form within their maternal cell, resulting in a “Russian doll”-like cellular morphology [67]. It is thus tempting to speculate that the demand for phospholipids is high during paramyxid development, which may have caused an expansion of genes involved in transmembrane lipid transport. In *B. ostreae*, gene gains were enriched for symporter activity, again reflecting the importance of well-developed transporter systems in these intracellular parasites.

Many Ascetosporea-specific gene clusters could not be confidently assigned GO terms (1436 out of 1992). While the functions of such clusters are difficult to determine, they may still be important to the parasitic lifestyle of Ascetosporea. Interactions with the host are important parameters for parasites and are driven by proteins expressed at the host-parasite interface, i.e., the plasma membrane and its immediate vicinity. Such proteins are known to encode a signal peptide (SP) which acts as a targeting signal for the secretory pathway [68]. Parasite cell surfaces are known to rapidly undergo evolutionary change [69], which has been suggested to be accompanied by expansions in gene families encoding SPs [61]. Hence, we searched for SPs in all proteins from Ascetosporea and the outgroup species. We found a significant enrichment of proteins with SP predictions in Ascetosporea-specific gene clusters (939 with SPs out of 7004 proteins), compared to gene clusters shared with one or more of the outgroup species (102 with SPs out of 6909 proteins; *p* < 10^−16^; Fisher’s exact test). Most of the proteins with SPs in Ascetosporea-specific gene clusters (893 out of 939) occurred in 676 of the clusters without GO annotations, thus hinting at some of the functions in these gene clusters. These results indicate that Ascetosporea have evolved novel classes of enzymes that may be secreted or targeted towards the cell surface, potentially having a role in host-cell interactions.

## Conclusions

Ascetosporea has long evaded comparative genomic study, with only recent efforts successfully sequencing and disentangling their genomes from those of the host and other organisms [27, 28]. Here, we more than double the number of available genomes for this enigmatic clade of parasites. While sequencing ascetosporean genomes is still highly challenging, as evidenced by our relatively fragmented genome assemblies, our results give a first glimpse into the diversity of ascetosporean genomes and their evolutionary relationships. Apart from numerous gene losses, we identified some expanded functional classes of ascetosporean proteins, which may have enabled their evolutionary success as parasites. Our results and data provide potential genic targets for development of control measures to limit the spread of these parasites in invertebrate populations.

## Methods

### Sample collection, culturing conditions, nucleic acid extraction, and sequencing

Data generation varied depending on type of sample and target species. Below we give a per-species summary.

The amoeba strain M6MM was isolated from the gills of finescale triggerfish, *Balistes polylepis* Steindachner, sampled in Mazatlan, Mexico, in July 2000. Gill clips were placed on malt and yeast extract seawater agar (MY75S in the Culture Collection of Algae and Protozoa, SAMS Ltd, Oban, UK) and the isolated amoebae were subsequently subcultured on the same agar type. The strain was passaged weekly by transferring agar slices with amoebae to fresh plates. All cultures were kept at 20 °C, in an incubator. Amoebae were cryopreserved in seawater containing 10% DMSO and stored in liquid nitrogen at the culture collection of the Institute of Parasitology, Biology Centre of Czech Academy of Sciences. For DNA/RNA extraction, amoebae were washed off the agar plates with seawater and centrifuged briefly. Pelleted amoebae were mixed with TNES buffer (4 M urea; 10 mM Tris–HCl, pH 7.5; 120 mM NaCl; 10 mM EDTA; 5% SDS), and DNA was extracted using a conventional phenol–chloroform extraction protocol [70]. For RNA extraction from pelleted amoebae, the Nucleospin RNA Kit (Macherey–Nagel) was used, following the manufacturer’s instructions. Sequencing libraries were prepared from 0.6 ng of DNA using the Chromium Genome reagent kit v2 (cat# 120257/58/61/62) according to the manufacturers’ protocol (#CG00043 Chromium Genome Reagent Kit v2 User Guide, 10X Genomics) and sequenced with paired-end 150 bp read length, NovaSeq 6000 system, SP flowcell, and v1 sequencing chemistry. For the RNA one sequencing library was prepared from 500 ng total RNA using the TruSeq stranded mRNA library preparation kit (cat# 20020595, Illumina Inc.) including polyA selection. Unique dual indexes (cat# 20022371, Illumina Inc.) were used. The library preparation was performed according to the manufacturers’ protocol (#1000000040498), and sequenced with paired-end 150 bp read length, MiSeq system, and v2 sequencing chemistry.

*Paramarteilia canceri* is a parasite of the velvet swimming crab (*Necora puber*). Details about the collecting protocols, animal dissections, and identification of individuals infected with *Paramarteilia canceri* are published in Collins et al. [20]. Shortly, the crabs were collected in March 2016 from Galway Bay, Ireland, and the different parts of tissues were stored in 99% ethanol. The diseased animals were detected with histology and PCR screening (see [20] for details). For each of the three samples, the DNA was isolated from two diseased and one healthy animal with QIAamp DNA Mini Kit (Qiagen) according to the manufacturer’s protocol with few exceptions. The tissues were removed from ethanol, and two tungsten carbide beads of 3 mm (from Qiagen) were added to each sample before insertion in liquid nitrogen. The frozen samples were disrupted with a TissueLyser II machine from Qiagen (1 min at 27 s/m). According to the protocol lysis buffer AL and RNase A (100 mg/ml, Qiagen) was added to each disrupted tissue sample and the mixture was incubated overnight at 56 °C in a slow shaker. For each DNA sample, sequencing libraries were prepared from 1 µg DNA using the TruSeq PCRfree DNA sample preparation kit (cat# 20015962, Illumina Inc.) and unique dual indexes (cat#20022370, Illumina Inc.), targeting an insert size of 350 bp. The library preparation was performed according to the manufacturers’ instructions (guide#1000000039279) with a paired-end 250 bp read length, NovaSeq 6000 system, SP flowcell, and v1.5 sequencing chemistry. The two DNA libraries of the diseased host tissues (FB67 (S2/16/18) and FB68 (S2/16/19)) were sequenced on one lane together with the *Bonamia ostrae* libraries (see below). The DNA library of the healthy host tissue (FB 69.2 (S2/16/12)) was sequenced on a separate lane.

*Marteilia pararefringens* was isolated from the blue mussel *Mytilus edulis.* Thirty specimens of blue mussels were collected in October 2018 at Agapollen, Bømlo municipality, Hordaland, western Norway (59° 50.4′ N, 5° 14.8′ E) and kept alive in containers with seawater during transport to Havforsk laboratory, Bergen. The animals were then individually sampled at the lab facility for digestive gland investigations by cytological imprints and biopsies dissected out for further processing and kept cold on ice in containers with 4 ml transport medium: EMEM medium [71] supplemented with antibiotics (penicillin, streptomycin). Further investigation was done at Veterinary Institute in Uppsala by microscopy of imprints stained with May Grünewald Giemsa and screened to determine the parasitic burden of *M. pararefringens.* Further processing and cell sorting followed according to the methodology by Robledo et al. [72]. Highly infected individuals were chosen for further cell enrichment and storage in − 70℃ as pelleted cell suspension confirmed with PCR. DNA was extracted from one of the highly infected mussels (sample FB21 (M18)) following the same protocol as for *Paramarteilia canceri*. RNA was isolated from the same tissue in two replicates (Mpar-FB82 and Mpar-FB83) with the Qiagen RNeasy Micro Kit following the manufacturers’ protocol. Sequencing libraries were prepared from 0.6 of ng DNA using the Chromium Genome Library preparation kit (cat# 120257/58/61/62) according to the manufacturers’ protocol (#CG00043 Chromium Genome Reagent Kit v2 User Guide), and sequenced on a HiSeqX, paired-end 150 bp read length, v2.5 sequencing chemistry. For the two RNA samples, the sequencing libraries were prepared from 500 ng of total RNA using the TruSeq stranded mRNA library preparation kit (cat# 20020595, Illumina Inc.) including polyA selection. Unique dual indexes (cat# 20022371, Illumina Inc.) were used. The library preparation was performed according to the manufacturers’ protocol (#1000000040498) and sequenced with paired-end 150 bp read length on a NovaSeq 6000 system, SP flowcell, and v1.5 sequencing chemistry. Sequenced on the same lane with *B. ostreae* RNA.

*Bonamia ostreae* was isolated from oyster juveniles (*Ostrea edulis*) surviving after a 9-month infection trial performed at the Centre for Environment, Fisheries and Aquaculture Science (Cefas), UK during 2019–2020. Healthy oysters used for the infection trials were originally from Lochnell oyster farm in Scotland, and these were infected with *Bonamia* that was isolated from oysters in Portsmouth Harbour, UK. After the infection trial, body parts of oyster juveniles were dissected out, pooled in a single 50-ml tube, kept cold, and shipped on ice to Uppsala for further processing at the National Veterinary Institute in February 2020. Pooled parts of oyster juveniles, five specimens per pool, were kept cold on ice, and homogenized by Ultra Turrax homogenizer. The cell suspension was filtered and concentrated by sequential filtration from 300 µm, 70 µm down to 30 µm using Pluristrainer filter caps placed on 50-ml filtration tubes according to published methodology [73]. The concentrated cell pellets were resuspended and further purified by discontinuous cell centrifugation on Percoll and finally purified on 20% sucrose cushion gradients following the same methodology [73]. Final pooled cell suspensions were pelleted and snap frozen in − 70℃, till further investigations. Additionally, a few specimens were directly frozen without processing as single-sample biopsies. DNA was isolated from the two types of infected tissue, a pooled *B. ostreae* cell suspension isolated from five diseased oyster juveniles (lab id FB64; 20-A016) and a diseased oyster juvenile (lab id FB74; U2002 14—0430/06). After three cycles of thawing and freezing at − 70℃, DNA was isolated using the Qiagen DNA blood and tissue kit and following the QIAamp DNA mini protocol.

### Genome assembly

The *M. mackini* raw sequencing data was generated previously [28, 74]. To make the datasets from different species as comparable as possible, we de novo assembled these data with the same methodology as for the newly sequenced genomes. Raw reads from all libraries that were sequenced using standard Illumina (i.e., not 10X Chromium) were adapter-trimmed using trimmomatic [75]. Next, we obtained genome assemblies from the healthy hosts as follows. *Necora puber*, the host of *Paramarteilia canceri*, was included in our sequencing runs and was de novo assembled using ABySS v. 2.3.4 [76] using default k-mer size. For *Ostrea edulis*, the host of *B. ostreae*, we assembled published data [77] using ABySS (*k* = 28). The resulting assembly size was smaller than expected (219 Mb versus ~ 2.2 Gb) [78], so we supplemented the dataset with a transcriptome assembly [79] (the full genome sequence had not yet been released at the time of analysis). The genomes of *Crassostrea gigas* and *Mytilus edulis*, the hosts *M. mackini* and *M. pararefringens*, respectively, were obtained directly from NCBI [80, 81] (GCA_902806645.1; GCA_019925275.1). The genome of *Paramikrocytos canceri* was retrieved from the European Nucleotide Archive (ERZ16272710), with the reasoning that it was sequenced and assembled previously by our group with highly similar methods [27].

To remove as much host data as possible from sequencing libraries targeting parasites, we mapped these libraries to their respective host genomes using BWA mem v. 0.7.17-r1188 [82], and unmapped reads were retained. These read sets were de novo assembled using SPAdes v. 3.15.3 [83], or in the case of 10X Chromium data, Supernova v. 2.1.1 [84]. The M6MM libraries were assembled directly using Supernova (since it was cultured, there was no need to remove any host sequences, even though bacterial sequences were removed according to the text below). For *Paramarteilia canceri*, the FB67 library yielded too little parasite data to be useful, and we proceeded with only the FB68 genome assembly. For *B. ostreae*, we pooled the two libraries (FB64, FB74) during genome assembly. Resulting contigs were investigated for remaining contaminants using Blobtools v. 1.1.1 [85], which plots GC content, sequencing coverage and taxonomic assignment of contigs based on Blast results (see Additional file 2: Fig. S2 for some examples). Either Blastn against NCBI nt or Diamond Blastx against NCBI nr was used (hits with e-value < 1E − 50 retained). Using these plots as a basis, contigs from probable contaminating organisms were identified. Next, we separated reads mapping to contigs from contaminants from the rest, and re-assembled the reads that did not map to contaminants using the same methods as before. Several runs of de novo assembly, contamination identification, and filtering were performed, until the remaining amount of identifiable contaminating sequence was negligible. The genomes sequenced using 10X Chromium technology (*M. pararefringens,* M6MM) were scaffolded using ARBitR v. 0.2 [86] based on BWA mem read mappings.

For quality assessment of resulting genome assemblies, we employed several complementary analyses and metrics. For technical assembly metrics, a custom script was used [87]. To investigate the correspondence between reads and cleaned assemblies, we used the *compare* module within KAT v. 2.4.2 [88]. For this purpose, we used the final read sets after cleaning out contaminants. For completeness assessment of conserved orthologs, we used the “genome” setting in BUSCO v. 5.2.2 [31] against the alveolata_odb10 database.

Our newly generated genome assemblies were searched for mitochondrial sequences the following way. From the annotated *P. brassicae* mitogenome [89], protein sequences were collected, and homologs were searched for using tBlastn (evalue < 1e − 5). This analysis revealed a 21-kb contig in *B. ostreae*, which additionally stood out by having lower GC content and higher read coverage than contigs describing the nuclear genome. A tiny (5 bp) overlap between the 5′ and 3′ ends of this contig indicated that it might be circular. We used MITOS2 [90] on Galaxy (https://usegalaxy.org/) to search for genes in this putative mitogenome. In the other species, no similar candidate contigs could be identified by the tBlastn analysis. Reasoning that mitogenomes could have been filtered out during the cleaning steps we took during genome assembly, we looked into our previously generated Diamond Blastx results for hits to cytochrome *c* oxidase. For this purpose, unclean genome assemblies, where only reads mapping to the host genomes had been removed, were used. In *M. pararefringens*, GetOrganelle [91] was used on the reads after removing host sequences, without generating any meaningful output. We also searched the nuclear genomes for homologs to POP; the plant and protist organellar DNA polymerase [36], using Blastp with our protein predictions as databases and the POP gene from *P. brassicae* (CEO96808) as query.

### Genome annotation

Structural and functional gene annotation was performed with funannotate v. 1.8.9 [92]. For each species, a de novo repeat library was created using RepeatModeler v. 2.0.2 [93] and repeats in the genome assemblies were softmasked with RepeatMasker v. 4.1.2-p1 [94]. Next, the genomes were annotated using the funannotate modules *clean, sort, train*, *predict*, *update*, and *annotate*, in that order. Default parameters were used except for adding –jaccard_clip during *predict.* Transcriptomic reads were used to train ab initio gene prediction software, except for *Paramarteilia canceri* (see below). In the case of *B. ostreae*, our transcriptome sequencing resulted in fewer parasite reads than expected, so instead we obtained the previously published *B. ostreae* transcriptomic raw data and used for annotation [33]. Before running *annotate*, functional annotations of predicted gene models were added using InterProScan-5.54–87.0 [95], Phobius [96], and eggNOG-mapper v. 2.1.5 [97]. BUSCO v. 5.2.2 [31] was used for completeness assessment of proteins compared to the alveolata_odb10 database.

For *Paramarteilia canceri*, no RNA sequencing data was available for training ab initio gene predictors, so we had to take another approach. First, repeats were annotated as above. Next, preliminary gene models were collected using ab initio predictors trained on *M. pararefringens* data. A subset of the resulting gene models was extracted for further training, based on the following criteria: a homolog of the model existed in at least three other Ascetosporea (excluding *B. ostreae*, which was not yet annotated at this time), and the model should not be present in more than one copy (paralog) in *Paramarteilia canceri*. For these purposes, orthology prediction was performed using OrthoFinder v. 2.5.2 [53]. The resulting training set consisted of 244 genes and was used to train Augustus v. 3.3.3 [98]. Trained Augustus models were used within funannotate for a second annotation run, including the other ab initio predictors within funannotate, followed by functional annotation as for the other species. Annotation statistics were collected for all genomes using GAG [99] and BEDTools [100]. The intergenic distance presented in Table 1 was calculated as the median distance between genes on the same scaffold.

During submission of annotated genomes to the NCBI genome database, the NCBI Foreign Contamination Screen [30] was automatically run for each genome. This pipeline identified some scaffolds that were of putative contamination origin, either from host or bacteria. These scaffolds, and the genes residing within them, were excluded from the submissions to this database. Note that this analysis took place at a late stage during manuscript preparation; hence, these genes were included in our analyses. However, since the number of putative contaminant genes was small, their impact on our results was deemed to be negligible.

### Phylogenomic dataset construction

To construct a phylogeny of Ascetosporea and related Rhizaria, we compiled a dataset based on previous work in our group [40, 41]. We extracted all rhizarian sequences from the full dataset from Schön et al. [41, 101] (excluding proteins from plastids, nucleomorphs, *Minchinia chitonis,* which was only represented by two genes in this dataset, and the previous *M. mackini* transcriptome). Furthermore, we extracted Stramenopiles, Alveolata, and *Telonema* from the reduced dataset from Schön et al. [41, 102]. *Filoreta tenera* (called *Corallomyxa* sp. in the previous dataset [24, 41]) had low gene representation in this dataset, and preliminary analyses placed M6MM as a close relative of *F. tenera.* To improve phylogenetic resolution around this clade, we sampled additional genes from *F. tenera*, by removing all its sequences from the previous dataset and adding proteins from the EukProt v.03 database instead [103]. To identify homologs in Ascetosporea, M6MM, and *F. tenera*, we searched their predicted proteomes with Blastp (keeping hits with > 50% query coverage and e-value < 1e − 20), using rhizarian homologs as queries (including previously identified *M. mackini* homologs). Matches were extracted and subjected to single-gene phylogenetic analysis with the full dataset from Schön et al. [101] (containing representatives from all major groups of eukaryotes and some prokaryotes). Putative homologs for each gene were aligned using MAFFT v. 7.310 (--auto to determine the alignment strategy), and gene trees were produced with IQ-TREE2, and FastTree v. 2.1.12 [104] within Geneious v 10.2.6 [105]. Gene trees were carefully reviewed for problematic sequences, in terms of paralogy, contamination, or low-quality gene annotations. Genes where the Blast analysis resulted in several hits, e.g., ribosomal proteins, were analyzed together in a combined tree to determine orthology. Fragmented genes were merged using a custom script [106].

### Phylogenomic analyses

After a high-confidence set of orthologs was obtained, sequences were aligned with MAFFT v. 7.310 (E-INS-I), trimmed using trimAl v. 1.2 (-gappyout), and concatenated [107]. The resulting supermatrix consisted of 225 genes and 67,786 amino acid positions. The matrix was subjected to ML phylogenetic analysis using IQ-TREE2 v. 2.2.0.3, using ModelFinder [43] internally to determine the best-fitting model of sequence evolution (resulting in LG + C60 + G + F; from testing all site-homogeneous LG models in addition to mixture models LG + C20-60 + G + F, and all combinations thereof). A thousand ultra-fast bootstrap replicates were sampled within IQ-TREE2. To minimize phylogenetic artifacts resulting from long branches, we removed all Ascetosporea except *B. ostreae* from the same dataset and re-ran the analysis with the same sequence evolution model as above. To investigate the effects of fast-evolving sites during rhizarian evolution, we performed incremental removal of such sites as follows. We used the script *fast_site_remover.py* within PhyloFisher v. 1.2.3 [108], with 7000-bp increments, resulting in nine sub-sampled datasets. From each subsampled matrix, a tree was built using IQ-TREE2 with the LG + C20 + G + F model, followed by another tree employing the posterior mean site frequency mixture model (PMSF) [109], using LG + C60 + G + F as input mixture model and the output tree from the C20 analysis as guide tree. From the sixth step of subsampling fast-evolving sites from the supermatrix (38% of sites remaining), we performed Bayesian phylogenetic inference with PhyloBayes-mpi v. 1.9 [44]. Three chains were run for > 7000 generations, discarding the first 700 generations as burn-in. A coalescent-based tree was constructed the following way. Single-gene trees were constructed from each of the 225 ortholog alignments, after merging fragmented genes but before trimming the alignments, using IQ-TREE2 v. 2.2.0.3 (-m TESTNEW -mset LG -bb 1000). ASTRAL-III v. 5.7.8 [110] was used with default settings to calculate a multi-species coalescent tree.

### Ancestral gene family reconstruction

We used Amalgamated Likelihood Estimation (ALE) [54] to model gene family evolution in Ascetosporea. The method uses reconciled gene trees to find events in the form of losses, duplications, originations, and transfers. We added the proteomes of *Reticulomyxa filosa*, *Plasmodiophora brassicae*, and *Bigelowiella natans* [50–52], to the analysis, for a total of nine species. These taxa were extracted from the supermatrix used for phylogenomic analysis above, and reduced-taxon tree with these taxa was created (IQ-TREE2, LG + C60 + G + F). Next, we used OrthoFinder to classify all predicted proteins from the nine species into orthogroups (OGs; or gene clusters). Proteins belonging to each cluster were aligned using MAFFT v.7.310, and 1000 ultra-fast bootstrap trees were created with IQ-TREE2 v.2.1.2 (-bb 1000 –boot-trees –bnni -m TEST). Clusters with three or fewer members, where trees could not be obtained, were counted as originations during the ALE analysis. To run ALE, a Nextflow pipeline was used [111], where the reduced-taxon species tree was specified, in addition to the ultra-fast bootstrap replicates of the single-gene trees. To reduce noise from the gene trees, events were counted only where they had a frequency higher than 0.3 [112].

### Comparative functional genomics

From the funannotate pipeline, Gene Ontology (GO) terms were mapped to the newly annotated genomes. To generate comparative datasets from the outgroup proteomes (*R. filosa*, *P. brassicae*, and *B. natans*), we applied InterProScan v. 5.54–87.0 [95], as wrapped within funannotate, to them. After GO terms had been annotated for all proteomes, the terms were mapped onto the OGs from OrthoFinder. To find GO term enrichment at specific branches in the reduced-taxon species tree, we applied GOATOOLS [113], where the “population” set consisted of all OGs where GO terms were mapped (excluding OGs without GO terms). At each branch in the tree, OGs with losses and OGs with duplications and/or originations were analyzed separately. Raw results of these analyses can be found in Dataset S3. After enriched GO terms were obtained, they were clustered based on semantic similarity using GO-Figure! [114].

All predicted proteins from the nine species were mapped onto major groups of metabolic pathways of the Kyoto Encyclopedia of Genes and Genomes (KEGG). This was performed using eggNOG-mapper v. 2.1.5 [97] with the v. 5.0 eggNOG orthology database. Signal peptides were predicted using SignalP v. 5.0 [115]. For the signal peptide enrichment analysis, all ascetosporean proteins were classified as shared with outgroups (*R. filosa, P. brassicae*, *B. natans*, or M6MM) or Ascetosporea-specific, based on OrthoFinder results. The number of proteins predicted to encode signal peptides was counted in the two categories, and Fisher’s exact test was performed using the R programming language.

### Supplementary Information

**Additional file 1: Figure S1.** Cleaning of ascetosporean metagenomes. Blobplots [85] of genome assemblies before (top row) and after (middle and bottom rows) cleaning of contaminant data. In each blobplot, contigs are represented as circles, size scaled to contig length, and with the color showing putative taxonomic affiliation. Blast hits to Rhizaria and other protists are represented in the “Eukaryota-undef” group. For the top two rows, taxonomy was based on Diamond Blastx searches against the NCBI non-redundant protein database (nr), and for the bottom row, against a custom database of rhizarian proteomes (excluding the proteome of the same species; e-value < 1e-50 in all cases). Contigs are positioned according to their GC content (x-axis) and depth of sequencing coverage (y-axis). Note that prior to assembly of the uncleaned datasets, reads mapping to the host genomes of the parasites were excluded. The *Paramikrocytos canceri* assembly was decontaminated using the same procedure, see Onut-Brännström *et al*. [27] for details.

**Additional file 2: Figure S2.** K-mer comparisons between reads and assemblies. Each panel represents the comparison of shared 27-mers between the cleaned assembly and cleaned reads. The x-axis represents k-mer multiplicity, i.e. the number of times distinct k-mers occur in the reads, while the y-axis represents the total number of distinct k-mers. Black bars represent k-mers found in the reads but not in the assembly, red bars k-mers that occurred once in the assembly, purple twice, etc. In *Bonamia ostreae*, the low sequencing coverage of the parasite caused the peak to shift far to the left. In haploid genomes, a single peak is expected, while two peaks are expected in diploids. Note that the cleaned reads were used in the analysis, so only a minor amount of data from the host and other organisms is expected. Plots created by KAT [88]. For the *Paramikrocytos canceri* genome, see Onut-Brännström *et al*. [27].

**Additional file 3: Figure S3.** BUSCO [31] results of A. genome assemblies and B. predicted proteomes of the six species in this study against the Alveolata ODB10 database.

**Additional file 4: Figure S4.** Blobplot of the publicly available *Bonamia ostreae* transcriptome [33]. Taxonomic affiliation of transcripts was determined based on a Diamond Blastx search against the NCBI non-redundant protein database (nr). For stringency, only Blast hits with an identity score >90% were retained. Note the clouds of transcripts annotated as Proteobacteria and Mollusca, indicating remnant contamination from these taxa.

**Additional file 5: Dataset S1.** Annotation of the putative *Bonamia ostreae* mitogenome. The annotation was automatically generated by MITOS2.

**Additional file 6: Dataset S2.** Phylogenomic dataset. In some cases, data were merged from multiple sources into OTUs (referred to as “merged” here). The full dataset is available in FigShare [117].

**Additional file 7: Figure S5.** A. Ultra-fast bootstrap support at selected branches after incremental removal of fast-evolving sites. Apofilosa refers to the branch grouping *Gromia*, *Filoreta*, and M6MM in a monophyletic clade, but not the topology therein. Ascetosporea includes the five analyzed species in this paper. The plus sign denotes sister relationship between the groups in question. The y-axis shows ultra-fast bootstrap support extracted from the C60 PMSF tree at each step. The subset of the supermatrix after six steps of fast-site removal (38% sites remaining) was selected for Bayesian analysis. B. Resulting consensus tree from three converged PhyloBayes [44] chains (>7,000 generations; 700 burn-in; CAT+GTR).

**Additional file 8: Figure S6.** Maximum likelihood tree (LG+C60+G+F) where long-branch Ascetosporea (*Marteilia pararefringens*, *Paramarteilia canceri*, *Paramikrocytos canceri*, *Mikrocytos mackini*) were excluded.

**Additional file 9: Figure S7.** ASTRAL tree. The tree was computed from single-gene trees of the 225 genes in our phylogenomic dataset using ASTRAL-III [110].

**Additional file 10: Dataset S3.** Full dataset of Gene Ontology (GO) term enrichment analysis at each branch within Ascetosporea. GO term enrichment analysis was performed on gene losses and gene gains (duplications and originations together) at each branch, and significant terms (*p* < 0.05) were retained.

**Additional file 11: Dataset S4.** Number of genes per genome with the “cilium” Gene Ontology term (GO:0005929). Only one isoform per gene model was counted.

## Data Availability

The data supporting the conclusions of this article are available in the NCBI GenBank database under the BioProject PRJNA1051017 [116]. Raw sequencing data can be accessed at the NCBI Sequencing Read Archive and annotated genome assemblies in the NCBI genome database. Phylogenomic datasets and the *N. puber* metagenome assembly are available in the FigShare repository [117].

## Abbreviations

ALE: Amalgamated Likelihood Estimation
CA: Common ancestor
GO: Gene ontology
KEGG: Kyoto Encyclopedia of Genes and Genomes
ML: Maximum likelihood
OG: Orthogroup
POP: Plant and protist organellar DNA polymerase
SP: Signal peptide
