# Supplementary figures and images for: Comparative genomics of Ascetosporea gives new insight into the evolutionary basis for animal parasitism in Rhizaria
Source: BMC Biol. 2024 May 3;22:103. doi: 10.1186/s12915-024-01898-x (PMC11069148; doi:10.1186/s12915-024-01898-x)

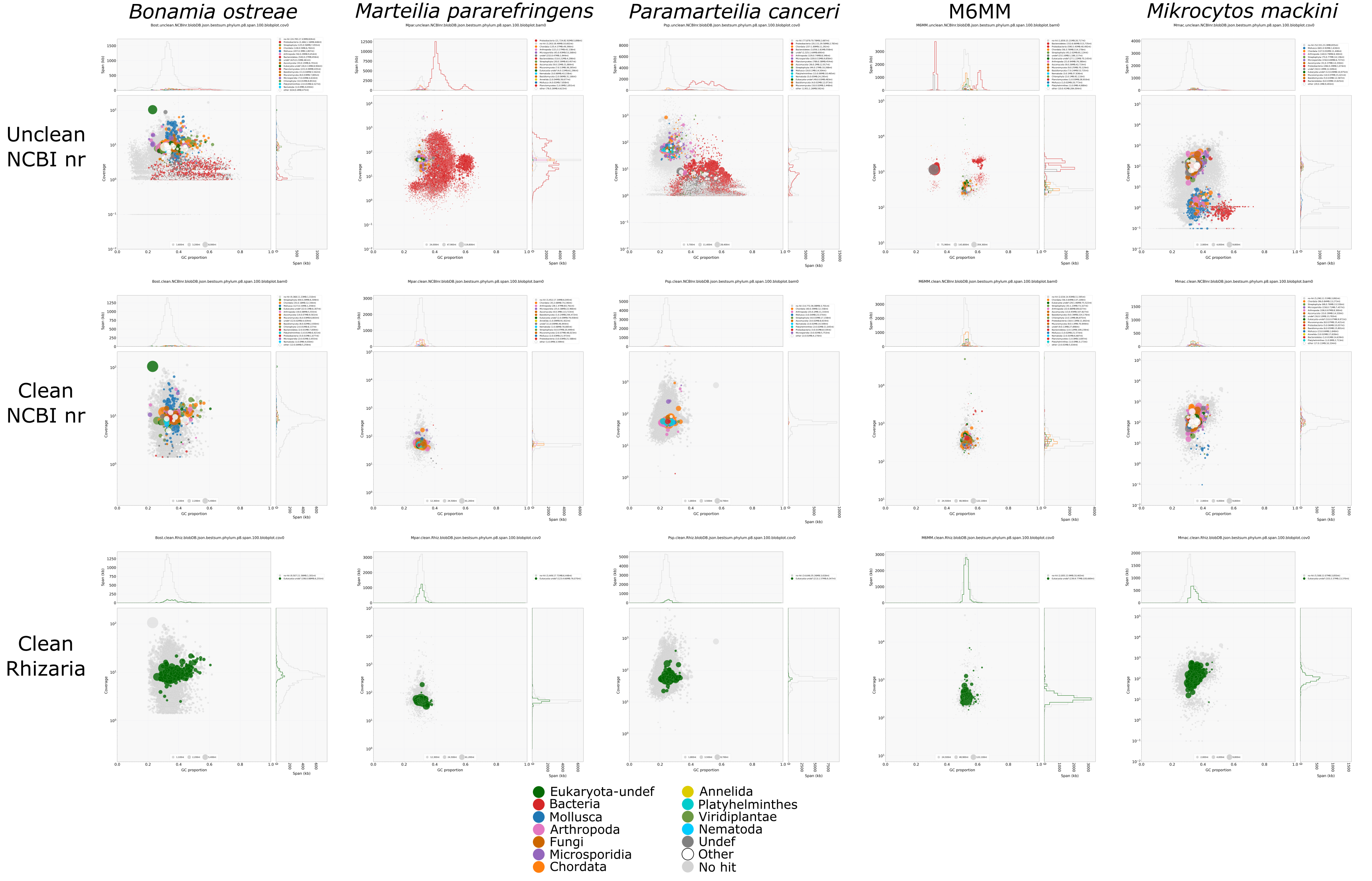

Supplement: Supplementary file 1 — Additional file 1: Figure S1. Cleaning of ascetosporean metagenomes. Blobplots [85] of genome assemblies before (top row) and after (middle and bottom rows) cleaning of contaminant data. In each blobplot, contigs are represented as circles, size scaled to contig length, and with the color showing putative taxonomic affiliation. Blast hits to Rhizaria and other protists are represented in the “Eukaryota-undef” group. For the top two rows, taxonomy was based on Diamond Blastx searches against the NCBI non-redundant protein database (nr), and for the bottom row, against a custom database of rhizarian proteomes (excluding the proteome of the same species; e-value < 1e-50 in all cases). Contigs are positioned according to their GC content (x-axis) and depth of sequencing coverage (y-axis). Note that prior to assembly of the uncleaned datasets, reads mapping to the host genomes of the parasites were excluded. The Paramikrocytos canceri assembly was decontaminated using the same procedure, see Onut-Brännström et al. [27] for details. [file 12915_2024_1898_MOESM1_ESM.png]

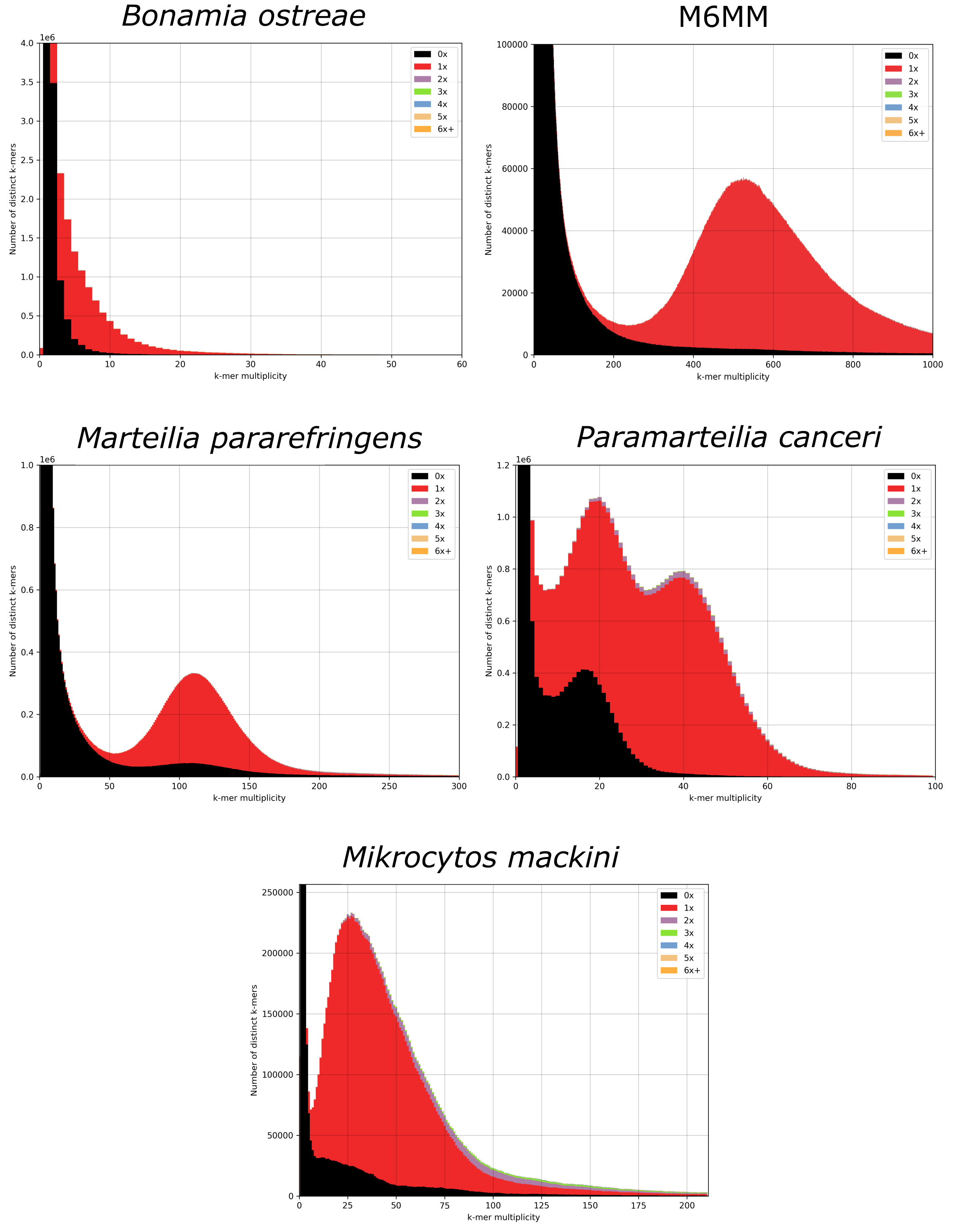

Supplement: Supplementary file 2 — Additional file 2: Figure S2. K-mer comparisons between reads and assemblies. Each panel represents the comparison of shared 27-mers between the cleaned assembly and cleaned reads. The x-axis represents k-mer multiplicity, i.e. the number of times distinct k-mers occur in the reads, while the y-axis represents the total number of distinct k-mers. Black bars represent k-mers found in the reads but not in the assembly, red bars k-mers that occurred once in the assembly, purple twice, etc. In Bonamia ostreae, the low sequencing coverage of the parasite caused the peak to shift far to the left. In haploid genomes, a single peak is expected, while two peaks are expected in diploids. Note that the cleaned reads were used in the analysis, so only a minor amount of data from the host and other organisms is expected. Plots created by KAT [88]. For the Paramikrocytos canceri genome, see Onut-Brännström et al. [27]. [file 12915_2024_1898_MOESM2_ESM.png]

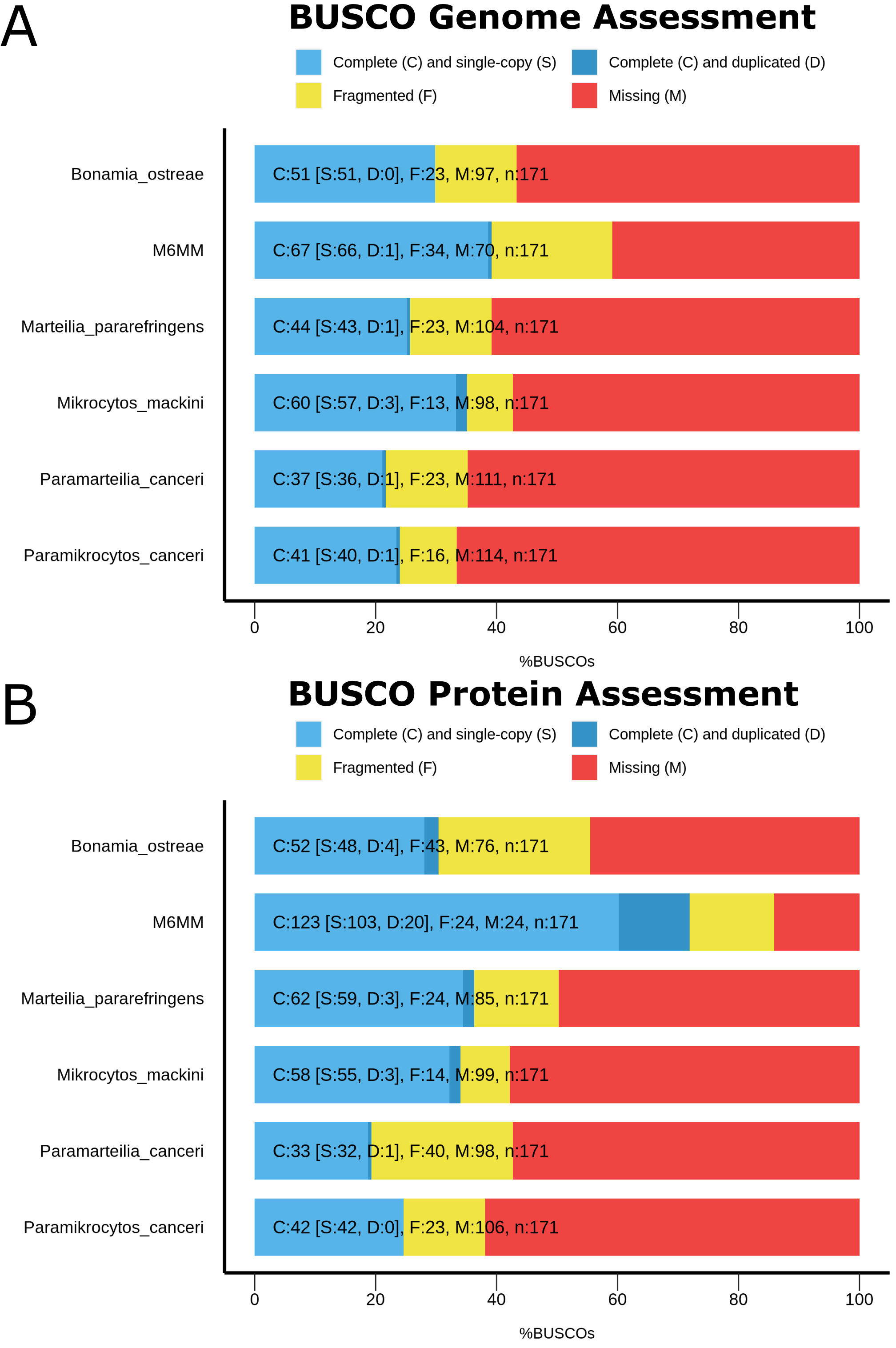

Supplement: Supplementary file 3 — Additional file 3: Figure S3. BUSCO [31] results of A. genome assemblies and B. predicted proteomes of the six species in this study against the Alveolata ODB10 database. [file 12915_2024_1898_MOESM3_ESM.png]

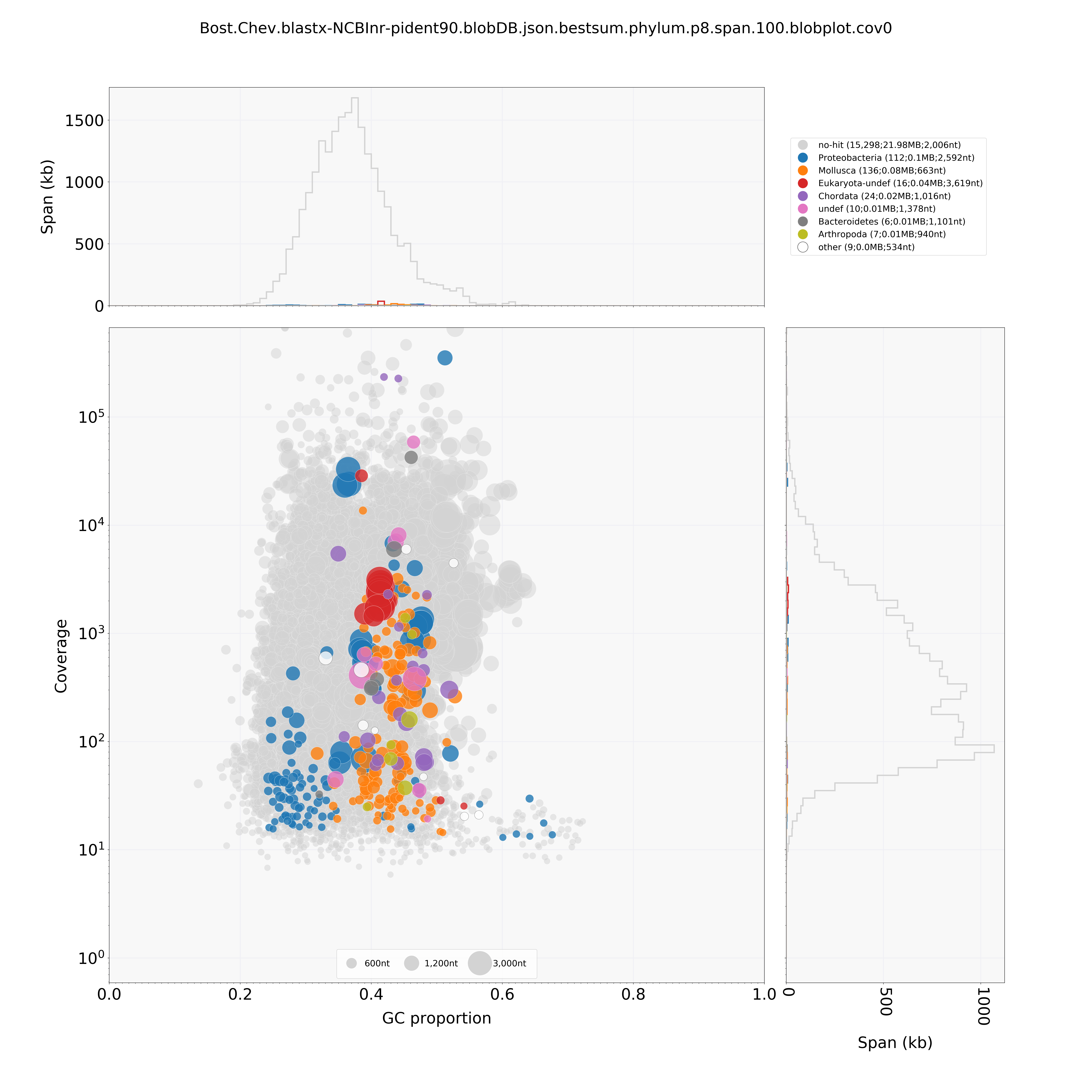

Supplement: Supplementary file 4 — Additional file 4: Figure S4. Blobplot of the publicly available Bonamia ostreae transcriptome [33]. Taxonomic affiliation of transcripts was determined based on a Diamond Blastx search against the NCBI non-redundant protein database (nr). For stringency, only Blast hits with an identity score >90% were retained. Note the clouds of transcripts annotated as Proteobacteria and Mollusca, indicating remnant contamination from these taxa. [file 12915_2024_1898_MOESM4_ESM.png]

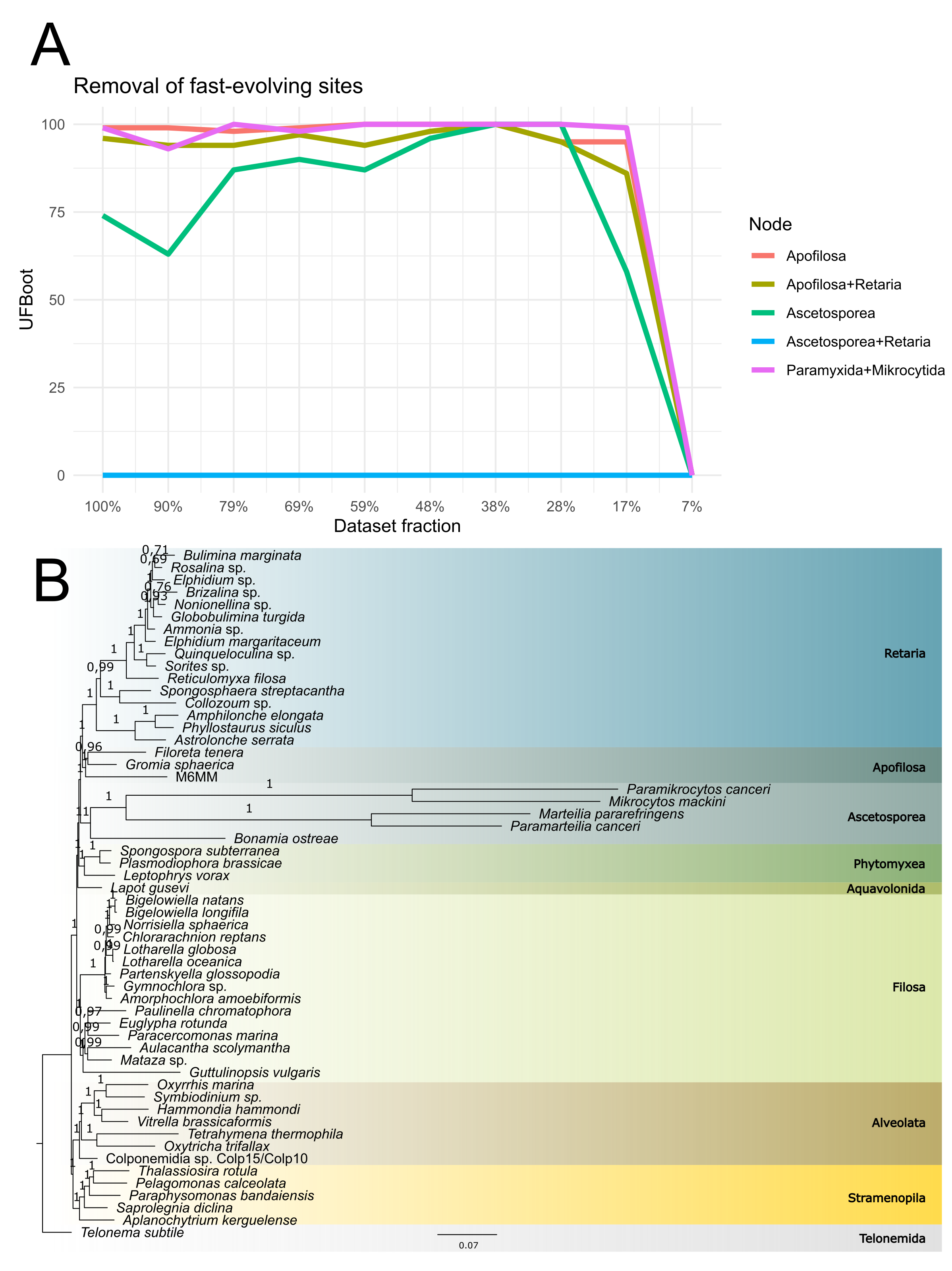

Supplement: Supplementary file 7 — Additional file 7: Figure S5. A. Ultra-fast bootstrap support at selected branches after incremental removal of fast-evolving sites. Apofilosa refers to the branch grouping Gromia, Filoreta, and M6MM in a monophyletic clade, but not the topology therein. Ascetosporea includes the five analyzed species in this paper. The plus sign denotes sister relationship between the groups in question. The y-axis shows ultra-fast bootstrap support extracted from the C60 PMSF tree at each step. The subset of the supermatrix after six steps of fast-site removal (38% sites remaining) was selected for Bayesian analysis. B. Resulting consensus tree from three converged PhyloBayes [44] chains (>7,000 generations; 700 burn-in; CAT+GTR). [file 12915_2024_1898_MOESM7_ESM.png]

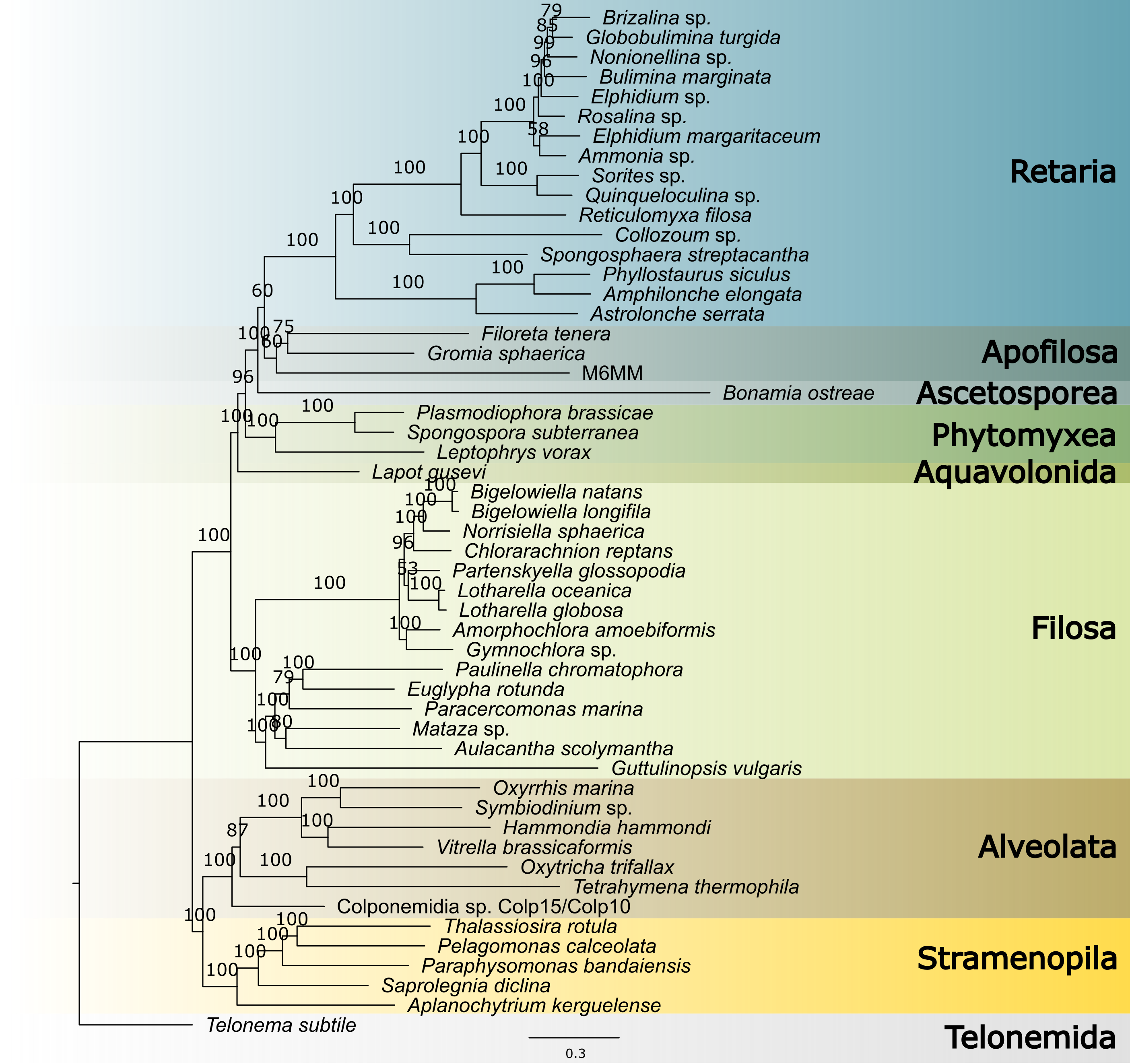

Supplement: Supplementary file 8 — Additional file 8: Figure S6. Maximum likelihood tree (LG+C60+G+F) where long-branch Ascetosporea (Marteilia pararefringens, Paramarteilia canceri, Paramikrocytos canceri, Mikrocytos mackini) were excluded. [file 12915_2024_1898_MOESM8_ESM.png]

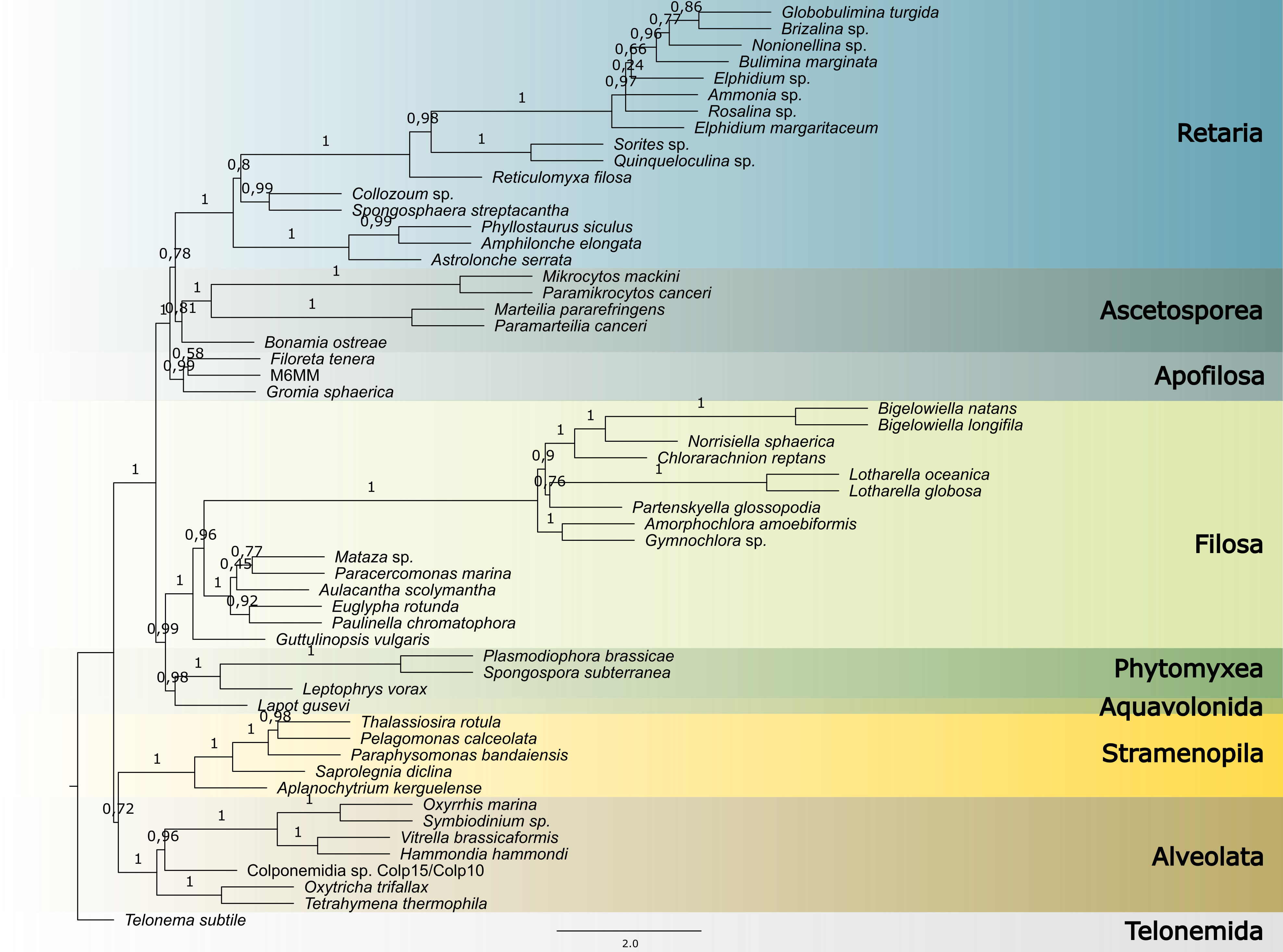

Supplement: Supplementary file 9 — Additional file 9: Figure S7. ASTRAL tree. The tree was computed from single-gene trees of the 225 genes in our phylogenomic dataset using ASTRAL-III [110]. [file 12915_2024_1898_MOESM9_ESM.png]
